# Thyroid metastasis from lung adenocarcinoma with EML4-ALK rearrangement

**DOI:** 10.1136/bcr-2016-217541

**Published:** 2016-11-21

**Authors:** Hironori Kawamoto, Yugo Kaneko, Kai Ryu, Kazuyoshi Kuwano

**Affiliations:** 1 Department of Respiratory Medicine, Tokyo Jikeikai Ika Daigaku Fuzoku Daisan Byoin, Komae, Japan; 2 Department of Respiratory Medicine, Tokyo Jikeikai Ika Daigaku, Minato-ku, Japan; 3 Division of Respiratory Diseases, Department of Internal Medicine, The Jikei University Daisan Hospital, Tokyo, Japan; 4 Tokyo Jikeikai Ika Daigaku Fuzoku Daisan Byoin, Komae, Japan; 5 Department of Respiratory Medicine, Jikei University School of Medicine, Tokyo, Japan

## Abstract

Thyroid metastases from lung cancer are very rare. A woman aged 42 years with a tumour in the lower lobe of the right lung was diagnosed as having lung adenocarcinoma positive for echinoderm microtubule-associated proteinlike 4-anaplastic lymphoma kinase. Positron emission tomography demonstrated fluorodeoxyglucose accumulation in the lower lobe of the right lung, the right thyroid lobe and both adrenal glands. We performed fine-needle aspiration biopsy (FNAB) and used reverse transcriptase-PCR (RT-PCR) to diagnose the patient as having metastatic lung adenocarcinoma to the thyroid gland. We believe that FNAB combined with RT-PCR can be an effective method for diagnosing metastatic lung adenocarcinoma to the thyroid gland.

## Background

Translocation of the anaplastic lymphoma kinase (ALK) gene echinoderm microtubule-associated proteinlike 4 (*EML4*) was shown in 5% of patients with lung adenocarcinoma.^1^ Crizotinib treatment has been reported to safely lead to a response rate of 60% and median progression-free survival of 8 months.^2^ ^3^

Thyroid metastases of non-thyroid malignancies have been reported in 1.4–3% of all thyroid malignancies.^4–9^ Thyroid metastases are most common in the kidney, colorectal, lung and breast cancer.^4^ ^10^ Clinically, the distinction between primary and secondary thyroid tumour is very important to determine staging and to treat the tumour.

We herein describe a patient diagnosed as having thyroid metastases with fine-needle aspiration biopsy (FNAB) combined with the reverse transcriptase-PCR (RT-PCR) method.

## Case presentation

A woman aged 42 years who had no relevant history and no history of cigarette smoking visited our hospital reporting of left inguinal pain for 1 month. Ultrasound examination revealed a left inguinal tumour of 60 mm in diameter. The pathological findings from the biopsy of the left inguinal lymph node showed poorly differentiated cancer. Metastasis from a gynaecological malignancy was suspected initially, but a gynaecological malignancy was not recognised.

**Figure 1:**
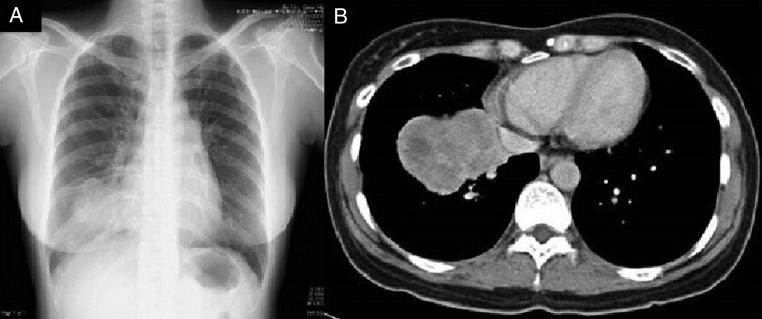
(A) Chest radiograph shows a mass in the right lower lung field. (B) Chest CT shows ∼70 mm mass in the right lower lobe. The border of the mass is irregular, and the interior is heterogeneous.

**Figure 2:**
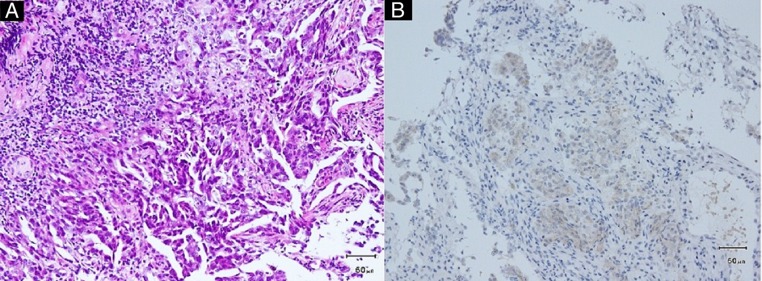
(A) Tumour cells produce a papillary structure. They partly show an irregular glandular cavity structure (HE stain, ×20). (B) Immunostaining for anaplastic lymphoma kinase demonstrates positive staining of the alveolar tissue.

**Figure 3:**
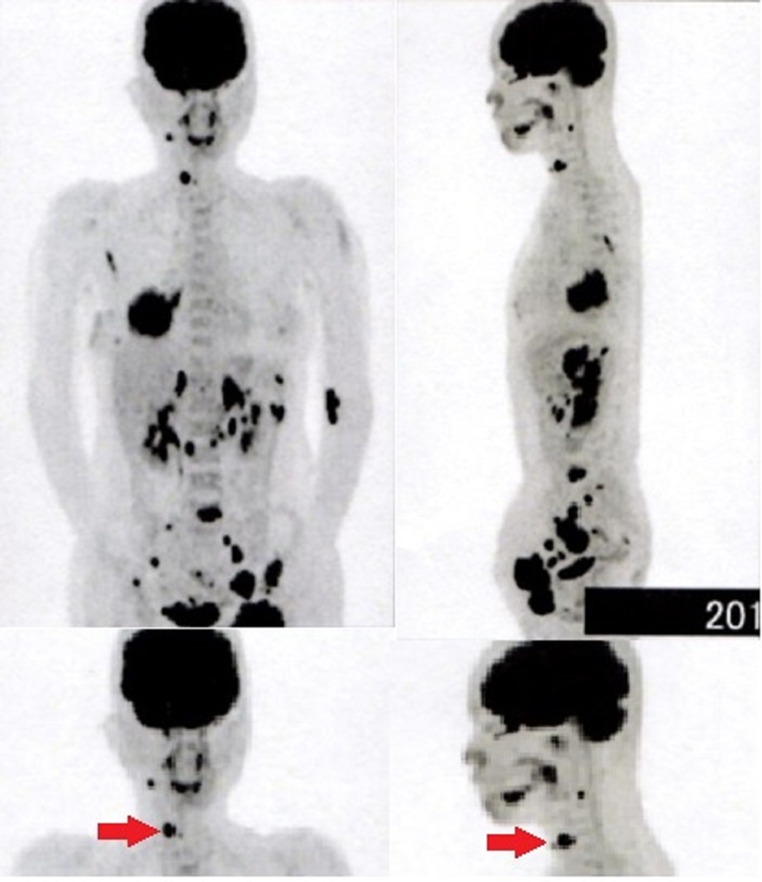
Positron emission tomography shows the highest standardised uptake values to be 14, 17 and 19, respectively, in the right lobe of the thyroid gland, the tumour in the right lower lung lobe and in the left adrenal gland. The right shoulder blade, the left ribs, lumbar vertebrae, the left ilium lymph node, the para-aortic lymph nodes, the bilateral total iliac artery lymph nodes, the left groin and the obturator lymph node also show fluorodeoxyglucose (FDG) accumulation. Arrows show FDG accumulation in the right thyroid lobe.

**Figure 4:**
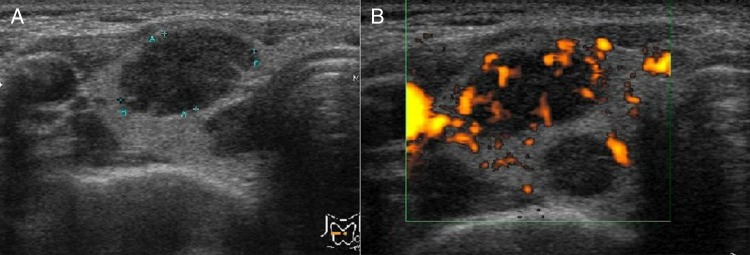
The thyroid ultrasound examination shows a tumour mass in the right lobe of the thyroid gland of ∼16 mm in diameter. Vacularisation is abundant within the mass.

**Figure 5:**
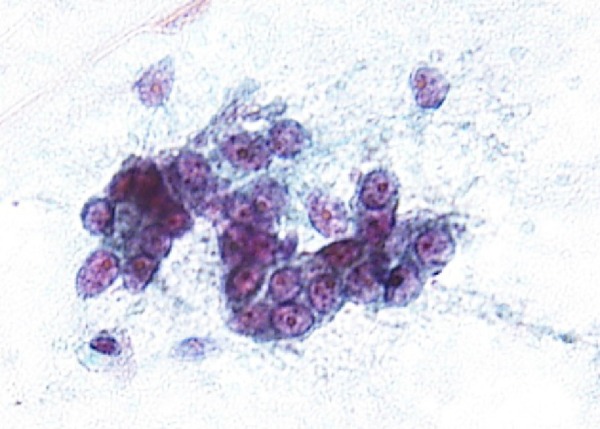
Malignant cells are present in the right lobe of the thyroid gland.

Chest X-ray and CT scan of the chest revealed a 70 mm tumour in the lower lobe of the right lung (figure 1A, B). The pathological findings of the transbronchial lung biopsy showed poorly differentiated adenocarcinoma with positive thyroid transcription factor-1 (TTF-1), carcinoembryonic antigen (CEA) and ALK by immunohistochemistry (IHC) (figure 2A, B). In addition, ALK positivity was revealed by the fluorescence in situ hybridisation (FISH) method, which indicated that 28% of the tumour cells showed either split red and green signals or single red signals. Afterwards, the metastasis of the left inguinal lymph node also was found positive for TTF-1 and ALK by IHC. Positron emission tomography (PET) demonstrated fluorodeoxyglucose (FDG) accumulation in the lower lobe of the right lung, the right thyroid lobe, both adrenal glands and other areas (figure 3). The highest standardised uptake value (SUV) in the right lobe of the thyroid gland was 14.0. We suspected thyroid metastasis from lung cancer and performed a thyroid ultrasound examination, which showed a hypervascular tumour of 16 mm in diameter (figure 4). We performed FNAB of the right lobe of the thyroid gland tumour to evaluate whether this was a primary tumour or metastasis (figure 5). The thyroid tumour was determined to be a metastasis from the lung adenocarcinoma because of the positive finding of EML4-ALK (variant 3a/3b was amplified) obtained using the RT-PCR method. We could not evaluate the thyroid tumour by IHC and FISH methods due to the smaller sample volume of FNAB. Multiple brain metastases in the left cerebellum were also observed with brain contrast-enhanced MRI. Therefore, we diagnosed the patient as having EML4-ALK-positive lung adenocarcinoma with a TNM classification of T3N1M1b, Stage IV. Her laboratory data showed normal thyroid function and a high CEA level (16.5 ng/mL).

**Figure 6:**
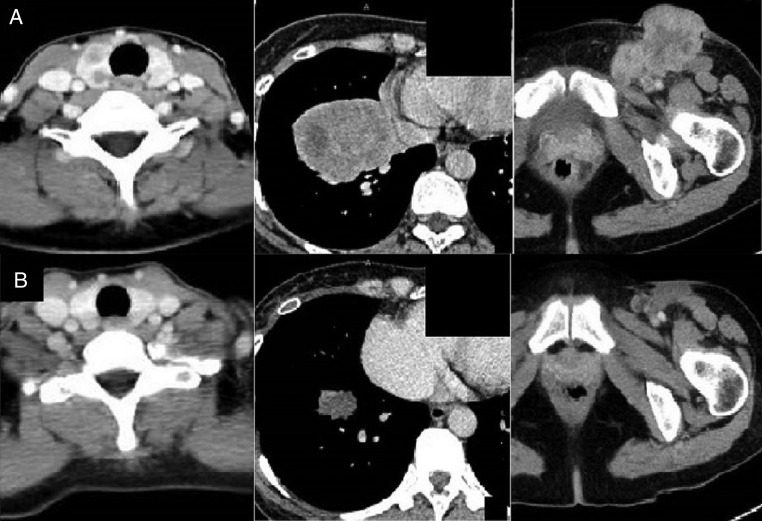
(A) CT shows the size in the right lobe of the thyroid gland, the right lower lung lobe and the left inguinal lymph node before treatment. (B) Three months after the beginning of crizotinib therapy. The tumour sizes are all reduced.

The multiple brain metastases in the left cerebellum were treated with stereotactic radiotherapy, because multiple brain metastases were at two places and tumours of 6 mm in maximum diameter. We initiated molecularly targeted drug therapy with crizotinib (500 mg/day). We judged a partial response 3 months after the beginning of treatment with crizotinib (figure 6A, B). The patient has been obtained effect under crizotinib treatment.

## Discussion

There have been only a few reports of thyroid metastases from lung cancer.^11^ ^12^ This is the first report, to the best of our knowledge, of thyroid metastases from lung adenocarcinoma with EML4-ALK rearrangement.

Generally, ALK is identified as tyrosine kinase target in non-small-cell lung cancer. ALK is aberrantly activated by chromosomal rearrangement or inversion that leads to the expression of an oncogenic fusion kinase, such as EML4-ALK. ALK is mutually exclusive and is a potential target for treatment.^1^ Patients with EML4-ALK-positive lung cancer are non-smokers and relatively younger^13–15^ and tend to be diagnosed at an advanced stage.^13^ ^15^

In 160 autopsied cases, Abrams *et al*^16^ reported rates of metastasis from lung cancer to the thyroid and inguinal lymph nodes of 4% and 1.3%, respectively.

Ho *et al*^17^ investigated 4281 cases in which PET was used to determine the initial stage in all malignant tumours. PET in 165 (4%) of these cases showed FDG accumulation in the thyroid gland. Only 4 of the 165 cases were proved to be malignant thyroid tumour. Further, they indicated that the average SUV of malignant thyroid lesions was significantly higher than that of benign lesions. However, it was impossible to identify an optimal cut-off for SUV to differentiate benign from malignant lesions. Even if PET shows FDG accumulation in the thyroid, cytological or histological verification is important to evaluate whether the thyroid tumour is benign or malignant.

EML4-ALK rearrangement can be identified by IHC, FISH or RT-PCR methods. IHC and FISH methods are generally reinforced by a screening test. The IHC method shows that the protein producing the EML4-ALK gene variation is stained by using various monoclonal antibodies. The advantage is simple comparatively. But the rates of specificity and sensitivity depend on the type of kit used and the staining strength and are lower than the RT-PCR method.^18^ ^19^ FISH and RT-PCR methods directly identify the genetic variation and detect EML4-ALK gene rearrangement. However, limitations of the FISH method are that it is expensive and requires a good fluorescence scope and technical expertise.^18^ In contrast, the advantages of the RT-PCR method is that it shows high specificity and sensitivity even when a smaller sample volume is collected.^18^ ^20^ However, the limitations of the RT-PCR method need attention because it cannot be detect minor variants of the EML4-ALK gene and it is necessary to acquire a fresh-frozen tissue sample for the extraction of RNA.^18^ ^20^

Most laboratories examine specimens obtained by FNAB to evaluate thyroid pathology. Thyroid FNAB is a less invasive method than biopsy. Good-quality RNA can be successfully isolated in about 98% of patients with thyroid FNAB.^21^ We believe that thyroid FNAB is effective for demonstrating cytology or histology, and metastatic thyroid tumour from ALK-positive lung cancer can be diagnosed accurately by RT-PCR.

In conclusion, metastatic thyroid tumours from EML4-ALK-positive lung adenocarcinoma are relatively rare, so it is important to prove the thyroid tumour to be metastatic. FNAB combined with RT-PCR can be an effective method to diagnose metastatic thyroid tumour.

**Learning points**

- This is the first report, to the best of our knowledge, of thyroid metastases from non-small-cell lung cancer with EML4-ALK rearrangement.
- Fine-needle aspiration biopsy (FNAB) combined with reverse transcriptase-PCR can be an effective method to diagnose metastatic thyroid tumour even when FNAB collects a smaller sample volume.
- Even if positron emission tomography shows fluorodeoxyglucose accumulation in the thyroid, cytological or histological verification may be important to evaluate whether the thyroid tumour is benign or malignant.
